# Life expectancy and mortality of people with and without diabetes in Aotearoa | New Zealand: A national cohort study

**DOI:** 10.1371/journal.pone.0345892

**Published:** 2026-05-04

**Authors:** Francois Verster, Nicholas Bowden, Philip J. Schluter, Lynne Chepulis, Ryan G. Paul

**Affiliations:** 1 Department of Endocrinology, Waikato Hospital, Health New Zealand, Te Whatu Ora, Waikato, New Zealand; 2 Department of Paediatrics and Child Health, Ōtākou Whakaihu Waka, University of Otago, Otago, New Zealand; 3 Te Kaupeka Oranga, Faculty of Health, Te Whare Wananga o Waitaha, University of Canterbury, Christchurch, New Zealand; 4 School of Clinical Medicine, Primary Care Clinical Unit, University of Queensland, Brisbane, Australia; 5 Division of Health, University of Waikato, Waikato, New Zealand; 6 Waikato Regional Diabetes Service, Health New Zealand, Te Whatu Ora, Waikato, New Zealand; The Chinese University of Hong Kong, HONG KONG

## Abstract

**Background:**

Diabetes mellitus (DM) is a significant driver of excess morbidity and premature mortality, though few contemporary or locally-derived data quantify this impact on New Zealanders, particularly Māori and Pacific Peoples. This prospective, national population study examines the life expectancy (LE) for New Zealanders with and without DM, and characterises mortality rates.

**Methods:**

Data from 01/01/2015 to 31/12/2019 housed in the Integrated Data Infrastructure, including the Virtual Diabetes Registry and Health New Zealand Mortality Collection, were linked. Estimated remaining LE years were calculated using abridged period life tables using the Chiang method for those with type 1 diabetes (T1D), type 2 diabetes (T2D), and without DM. Sub-group analysis by ethnicity was completed. Five-year cause-specific mortality rates per 100,000 people were compared by DM group.

**Findings:**

T1D and T2D prevalence was 0.4% and 5.5% in the overall sample (*n* = 4,505,478). Considerable ethnic differences in LE were evident, for example a 13-, 17.1-, and 24.5-year loss in remaining LE years was seen in men aged 0–19 years with T1D in the overall, Māori, and Pacific Peoples groups respectively compared to those without DM. An age-dependent reduction in remaining life expectancy years was also evident; for example an 8.6 (8.5–8.7) year reduction seen in females with DM aged 0–4, and 4.0 (4.0–4.1) year reduction for women with DM aged 65–69. Those with DM had higher cause-specific death rates.

**Interpretation:**

DM is associated with age-dependent reductions in LE across the entire lifespan, with disproportionately higher LE loss seen in Māori and Pacific Peoples.

## Introduction

Despite improvements in diabetes-related mortality and life expectancy (LE) observed internationally, diabetes mellitus (DM) remains a major driver for excess morbidity and premature mortality [1–7]. In the Aotearoa | New Zealand (AoNZ) healthcare setting, there are several limitations to the available data describing the LE and cause-of-death effects of DM. Furthermore, while NZ Europeans with DM are arguably comparable to some international cohorts, there is a scarcity of data specifically describing LE outcomes for Māori (indigenous New Zealanders) and Pacific Peoples (the majority of whom have now been born in AoNZ) with DM. This is particularly notable given that Māori and Pacific Peoples living in NZ continue to be over-represented with regards to DM prevalence, relative inequalities, and adverse outcomes, even when adjusting for numerous confounders, and are under-represented in global and national data reporting [8–12]. Despite the two- to four-fold higher burden of T2D in Māori and Pacific Peoples’ being described since the 1960’s, recent analyses continue to demonstrate higher hazard ratios for all-cause mortality and hospitalisation rates for Māori and Pacific Peoples with DM [9,13].

Historical data for New Zealanders with T1D in the 1980’s estimated an estimated LE loss of 16.5 years, although this dataset was highly limited geographically and treatment has clearly evolved since then [14]. An international 2022 study estimated 10 years of life lost for New Zealanders diagnosed with T1D at age 10 years, though employed a modelling approach to provide country-specific estimates [15]. A recent report estimated the premature death of 54,000 New Zealanders aged under 85 years with type 2 DM (T2D), but did not analyse specific age or ethnic groups, did not provide exact quantification of LE loss, and did not provide comparison to T1D [16].

In keeping with trends described globally, it is recognised that the majority of New Zealanders with diabetes will die from cardiovascular disease, and that diabetes will increase the risk of death from other causes such as solid malignancies, renal disease and suicide [7,17–20]. However, the exact distribution of causes and rates of death in New Zealanders with diabetes are not known, nor whether there are any differences between T1D and T2D.

Given these gaps in contemporary AoNZ data regarding DM and its mortality-related effects, we have examined the effect on LE for New Zealanders living with DM compared to the general population using data from the Integrated Data Infrastructure (IDI), including the NZ Virtual Diabetes Registry (VDR) and Health New Zealand (HNZ) Mortality databases. Given persisting inequities, there was a specific focus on Māori and Pacific Peoples’ outcomes. As a secondary analysis, we also characterise the causes and rates of mortality for New Zealanders with DM.

## Methods

### Project registration and ethics

Health and Disability Ethics Committee (HDEC) approval was sought and but did not meet the threshold for formal ethics committee review based on the outcome of the HDEC checklist process. Access to the IDI for the study was approved by Statistics New Zealand (reference: MAA2016−59), with access to the data being under conditions that satisfy the security and confidentiality provisions of the Statistics Act (1975). Informed consent was not required as per legislation in the Statistics Act (1975). Confidentiality rules for Statistics New Zealand also required random rounding of counts to base three, or suppression if the raw count was less than 6, as has been applied in this study. This project was also registered with the Health New Zealand (HNZ) (Waikato) Research Office (RD024035) and approved by the Te Puna Oranga Māori Research Review Committee.

### Study design

A population-based, record linkage prospective cohort design was undertaken and included all individuals alive in the AoNZ estimated resident population (ERP) as at 31/12/2014 using the IDI. Participants were followed up over a 5-year period from 01/01/2015 to 31/12/2019 or until death, whichever came first. Data were accessed for research purposes from 24/07/2024 until 14/04/2025. Participants were excluded if their age was missing or outside the 0–110 year age range. The IDI is a large database derived from de-identified government, non-government, and survey data and links data at individual and household levels using a central linking dataset known as the IDI spine [21]. The linkage is primarily achieved through probabilistic matching of identifying information (for example name, date of birth, sex, and address), which is removed before researchers access the de-identified data [22]. The ERP is based on census data but also factors in residents not in AoNZ at time of census, as well as adjusting for anticipated birth and death rates between the census date and the applied study date [23,24]. Participants with DM were identified using the VDR, a national register of all New Zealanders with DM that is derived from health data such as use of hospital services, lab testing results, and pharmaceutical dispensing [25,26]. Since the VDR does not explicitly code for T1D or T2D, previously-published algorithms were applied to the VDR to identify those with T1D versus T2D [27,28]. Age-specific mortality rates (by 5-year intervals), stratified by sex, ethnicity and DM status, were calculated using HNZ mortality data which, when applicable, was linked to the VDR via anonymised identifiers.

### Mortality

Mortality was determined using the HNZ Mortality Collection [29]. All deaths among the cohort, and cause-specific mortality as recorded on the mortality records, were extracted with the proxy date of death set as the 15th day of the recorded month and year of death (exact dates are not available due to Statistics New Zealand privacy restrictions). Causes of death were classified by ICD chapter using the WHO International Classification of Diseases 10th Revision (ICD-10) [30]. Malignant neoplasms were further segregated into solid (ICD-10 C00 - C86, and C88.4) and liquid (ICD-10 C90 - C96) categories. Death resulting from coma and/or ketoacidosis for T1D and T2D were clustered together (ICD-10 E10.0 + E10.1 for T1D and E11.0 + E11.1 for T2D). Diabetic nephropathy for all people with diabetes was aggregated by combining categories E10.2, E11.2, E12.2, E13.2, and E14.2. Finally, circulatory disease-related causes of death had further subgroups created for ischaemic heart disease (ICD-10 I20 - I25) and cerebrovascular disorders (ICD-10 I60 - I69).

### Sociodemographic variables

Sociodemographic variables for the participants, including sex, age, and ethnicity were captured from the personal details tables of the IDI [23,31]. For participants identifying with multiple ethnicities, level 1 prioritisation was applied [32]. Given the anticipated small proportion of those identifying as Middle Eastern, Latin American, or African (MELAA), these individuals were combined into the European/Other group used in subsequent analyses. Geographic variables, including area level deprivation, health care region, and urban/rural profile of residence, were determined using IDI address history data to identify the meshblock of residence (a small geographic unit representing a typical neighbourhood size) for each individual on 31 December, 2013. Deprivation level was ascertained using the New Zealand Index of Deprivation Index 2013 (NZDep). NZDep is an area level measure of socioeconomic deprivation derived from multiple census domains such as income, employment, home ownership, and education level [33]. NZDep was collapsed into quintiles (quintile 1 least deprived, quintile 5 most deprived). Health care region was determined by the District Health Board (DHB) that the address was located within. Urban/rural profile of residence was based on the Statistics New Zealand definition and collapsed into a binary measure reflecting urban and rural areas [34].

### Statistical analysis

Estimated remaining LE, defined as the average years of life remaining, was calculated using abridged period life tables constructed using the Chiang method by 5-year intervals up to age 100 years, and an open-ended interval thereafter [35]. Life tables were constructed for the total population, with separation by sex, and with sub-group analysis by level 1 ethnicity. Due to the small number of observed events in some subgroups, 20-year age bands up to 80 years (and open interval thereafter) were employed for ethnic-specific remaining LE years analyses. For the secondary analysis exploring all-cause mortality, rates per 100,000 people were calculated and stratified by the above-described ICD-10 categories and by DM group. Given this was a secondary analysis, observed results only, and no inferential statistics, are presented.

Data analysis was carried out using Stata MP version 16.1 (StataCorp LLC, College Station, TX, USA) [36].

## Results

The ERP on 31 December 2014 contained 4,505,613 people. After restricting to men and women aged 0–110 years, the analytical sample included 4,505,472 people, of which 17.6% were Māori and 6.8% were Pacific Peoples. At the end of the five-year study period (31 December 2019), the study captured 22,140,240 person-years of data. For the overall population, there was a minor female predominance (50.4%). The median age at study entry was 37 years (IQR 18–55 years), with 16.2% aged 60–79 years, and 3.6% aged ≥80 years. The median age for those with DM was 62 years (51–73 years) and 35 years (17–53 years) for those without DM. The majority of people within the European/Other category were identified as European, with only 87,036 (3.0%) of participants of the 2,862,099 in this group being ‘Other’. Full sociodemographic characteristics are outlined in Table 1.

**Table 1:** Sociodemographic characteristics of the total population (restricted between 0–110 years).

	n	(%)
*Sex*		
Male	2,235,207	(49.6)
Female	2,270,265	(50.4)
*Age groups (years)*		
0-19	1,206,054	(26.8)
19-39	1,194,645	(26.5)
40-59	1,212,483	(26.9)
60-79	730,968	(16.2)
80+	161,328	(3.6)
*Ethnicity*		
Māori	790,968	(17.6)
Pacific Peoples	305,826	(6.8)
Asian	546,582	(12.1)
European/other	2,862,099	(63.5)
*Level of deprivation* ^a^		
1 (least deprived)	915,126	(20.6)
2	880,746	(19.8)
3	867,873	(19.6)
4	869,736	(19.6)
5 (most deprived)	903,972	(20.4)
*Residential region* ^b^		
Urban	3,872,559	(86.9)
Non-urban	581,322	(13.1)

^a^68,019 (1.5%) missing

^b^51,591 (1.1%) missing

^c^Total population in Table 1 vs Table 2 differs due to rounding to base 3

As described in Table 2, the overall sample included 264,048 (5.9%) people with diabetes; 15,939 (0.4%) diagnosed with T1D and 248,112 (5.5%) with T2D. Of note, mortality rates in Table 2 are unadjusted and, as such, do not account for differences in age distribution by ethnicity. In men and women, 5.9% and 5.8% had a diagnosis of DM respectively. Māori men and women had a comparable prevalence of T1D (0.2% vs 0.3% respectively) and T2D (5.3% vs 5.6%), as well as unadjusted 5-year mortality rates (2.5% vs 2.2%). European/other men and women had a similar prevalence of T1D (0.4% for both) and T2D rate (5.1% and 4.7%) though overall 5-year mortality rates were nearly twice as high in comparison to Māori (4.5% and 4.4%). T2D prevalence was highest in Pacific Peoples for both men and women (8.7% and 10.1% respectively).

**Table 2:** DM prevalence and overall 5-year mortality, stratified by ethnicity and sex.

	Total	T1D		T2D		Total Deaths^a^	
Ethnicity	N	n	(%)	n	(%)	n	(%)
*Men*							
Māori	395,322	906	(0.2)	21,075	(5.3)	9,948	(2.5)
Pacific Peoples	154,128	216	(0.1)	13,446	(8.7)	3,120	(2.0)
Asian	270,942	327	(0.1)	18,153	(6.7)	2,673	(1.0)
European/Other	1,414,818	6261	(0.4)	72,279	(5.1)	63,399	(4.5)
Total	2,235,210	7,710	(0.3)	124,953	(5.6)	79,140	(3.5)
*Women*							
Māori	395,649	1,170	(0.3)	22,341	(5.6)	8,730	(2.2)
Pacific Peoples	151,698	375	(0.2)	15,315	(10.1)	2,712	(1.8)
Asian	275,640	696	(0.3)	17,868	(6.5)	2,193	(0.8)
European/Other	1,447,281	5,985	(0.4)	67,635	(4.7)	63,324	(4.4)
Total	2,270,268	8,226	(0.4)	123,159	(5.4)	76,959	(3.4)

a Raw mortality data not adjusted for age.

The distribution of diabetes diagnoses by age group as of 31 December 2014 is described in S1 Table. For the T1D population, prevalence in younger age groups was higher in comparison to T2D. For example, 15.4% vs 0.9% of men with T1D vs T2D respectively were diagnosed in the 0–19 years age band. In contrast, the proportion of those with T2D is much higher in older age bands, for example 49.3% and 44.2% in men and women respectively aged 60–79 years with T2D.

**Fig 1:**
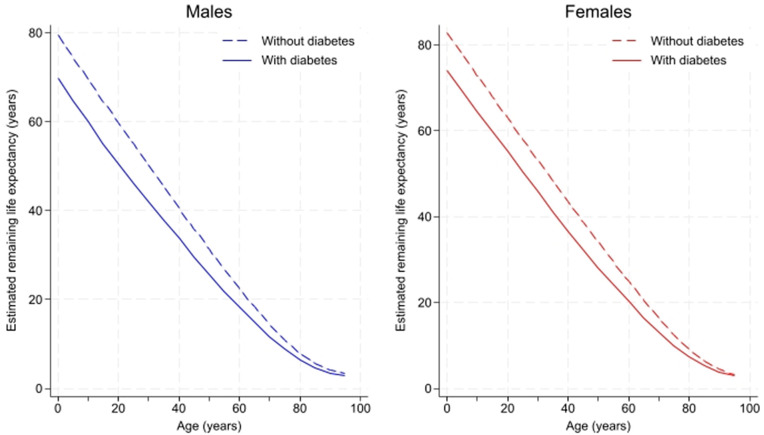
Estimated remaining life expectancy (years) by age for people with vs without diabetes.

Estimated remaining life expectancy years for individuals with any form of diabetes versus those without diabetes for the overall sample, stratified by sex, is presented in Fig 1. Full abridged period life table data, including confidence intervals, is found in S2 Table. At age 0–4 years, boys with diabetes have an estimated remaining LE of 69.7 years (95% CI: 69.6, 69.8), compared to 79.4 years (95% CI: 79.4, 79.4) for those without DM, resulting in a 9.7-year difference (95% CI: 9.6, 9.8). Girls with diabetes at age 0–4 years had a life expectancy of 74.2 years (95% CI: 74.1, 74.3) compared to 82.8 years (95% CI: 82.8, 82.8) for those without DM, representing an 8.6 years difference (95% CI: 8.5, 8.7). The life expectancy difference between those with and without DM decreased with increasing age; for example, at age 85–89 years, men with diabetes have a remaining life expectancy of 4.7 years (95% CI: 4.6, 4.7) compared to 5.7 years (95% CI: 5.6, 5.7) for those without diabetes.

Table 3 describes estimated remaining life expectancy for the total population and all study sub-groups, stratified by T1D or T2D, or those without DM, and by age group and sex. Across all categories, individuals without diabetes had a higher remaining LE than their age- and ethnicity-matched peers. Also, life expectancy for Māori without DM was shorter than for other ethnic groups without DM. Estimated life expectancies were higher among women compared to men across all ethnicities (for matched age groups), and estimated remaining LE was shortest for those with T1D across all matching age and ethnic groups. In terms of the absolute estimated remaining LE values, considerable ethnic differences were evident. For example, among men aged 0–19 years with T1D, the estimated remaining LE ranged from 54.7 years (95% CI: 51.8, 57.6) for those who identified as Pacific Peoples to 73.5 years (95% CI: 72.4, 74.6) for those who identified as Asian, representing a 18.8 year difference. Similarly, among women aged 0–19 years with T1D, the estimated LE ranged from 60.8 years (95% CI: 58.7, 63.0) for those who identified as Māori to 80.0 years (95% CI: 78.7, 81.3) for those who identified as Asian (a 19.2 year difference).

**Table 3:** Abridged period life table for T1D, T2D and no diabetes stratified by sex, age and ethnicity.

Cohort	Age Group (Years)	N	Estimated Remaining Life Expectancy (Years) (95% CI)		
			T1D	T2D	Without Diabetes
Overall Sample	Males				
	0-19	619,287	65.8 (64.7, 66.9)	67.6 (66.8, 68.5)	78.8 (78.5, 79.1)
	20-39	603,654	47.1 (46.9, 47.3)	48.7 (48.6, 48.8)	59.2 (59.2, 59.3)
	40-59	593,784	30.2 (30.0, 30.4)	32.1 (32.1, 32.2)	40.1 (40.1, 40.1)
	60-79	353,985	15.4 (15.3, 15.6)	17.2 (17.1, 17.2)	22.0 (22.0, 22.0)
	80+	64,500	4.1 (4.1, 4.1)	5.1 (5.1, 5.1)	7.1 (7.1, 7.1)
	Females				
	0-19	586,767	69.7 (68.8, 70.6)	71.5 (70.0, 72.9)	82.1 (82.0, 82.3)
	20-39	590,991	50.8 (50.6, 51.0)	53.4 (53.3, 53.4)	62.4 (62.4, 62.4)
	40-59	618,699	32.0 (31.8, 32.2)	35.1 (35.0, 35.1)	42.9 (42.9, 42.9)
	60-79	376,986	16.1 (15.9, 16.3)	18.9 (18.9, 19.0)	24.3 (24.3, 24.3)
	80+	96,828	4.4 (4.4, 4.4)	6.0 (6.0, 6.0)	8.3 (8.3, 8.3)
Asian	Males				
	0-19	75,645	73.5 (72.4, 74.6)	80.3 (80.2, 80.5)	89.2 (89.0, 89.3)
	20-39	113,604	53.5 (52.4, 54.6)	60.3 (60.2, 60.5)	69.4 (69.3, 69.5)
	40-59	57,456	35.0 (34.0, 36.1)	41.5 (41.4, 41.6)	49.8 (49.7, 49.8)
	60-79	21,888	18.6 (17.7, 19.4)	23.8 (23.7, 23.9)	30.9 (30.8, 30.9)
	80+	2,349	6.3 (6.3, 6.3)	9.4 (9.4, 9.4)	14.8 (14.8, 14.8)
	Females				
	0-19	70,668	80.0 (78.7, 81.3)	80.8 (78.0, 83.7)	92.8 (92.7, 92.9)
	20-39	107,043	60.6 (59.3, 61.8)	65.0 (64.9, 65.1)	72.9 (72.9, 73.0)
	40-59	69,690	40.6 (39.4, 41.8)	45.3 (45.3, 45.4)	53.2 (53.1, 53.2)
	60-79	25,341	21.2 (20.0, 22.4)	26.7 (26.6, 26.7)	34.1 (34.0, 34.1)
	80+	2,895	6.5 (6.5, 6.5)	11.2 (11.2, 11.2)	16.6 (16.6, 16.6)
European/Other	Males				
	0-19	313,008	67.1 (66.2, 68.0)	69.0 (68.2, 69.8)	79.1 (78.8, 79.4)
	20-39	334,269	48.2 (48.0, 48.5)	50.0 (49.9, 50.2)	59.5 (59.4, 59.5)
	40-59	417,867	30.9 (30.7, 31.1)	32.7 (32.7, 32.8)	40.2 (40.2, 40.2)
	60-79	290,808	15.8 (15.6, 15.9)	17.1 (17.1, 17.2)	21.9 (21.9, 22.0)
	80+	58,866	4.1 (4.1, 4.1)	5.0 (5.0, 5.0)	6.9 (6.9, 6.9)
	Females				
	0-19	296,727	70.9 (70.4, 71.5)	72.0 (70.3, 73.7)	82.3 (82.2, 82.5)
	20-39	329,013	51.6 (51.4, 51.9)	54.2 (54.1, 54.3)	62.5 (62.5, 62.5)
	40-59	427,077	32.7 (32.5, 32.9)	35.6 (35.5, 35.6)	42.9 (42.9, 43.0)
	60-79	306,135	16.5 (16.3, 16.7)	18.9 (18.8, 18.9)	24.2 (24.2, 24.2)
	80+	88,335	4.4 (4.4, 4.4)	5.6 (5.6, 5.6)	8.1 (8.1, 8.1)
Māori	Males				
	0-19	169,809	57.8 (56.3, 59.2)	60.6 (59.6, 61.7)	74.9 (74.5, 75.4)
	20-39	108,753	39.3 (38.6, 39.9)	41.8 (41.6, 42.0)	55.5 (55.4, 55.5)
	40-59	84,981	23.9 (23.3, 24.4)	26.5 (26.4, 26.6)	36.9 (36.9, 37.0)
	60-79	29,466	10.9 (10.7, 11.2)	14.3 (14.2, 14.4)	20.2 (20.1, 20.2)
	80+	2,310	2.8 (2.8, 2.8)	4.2 (4.2, 4.2)	7.1 (7.1, 7.1)
	Females				
	0-19	160,962	60.8 (58.7, 63.0)	65.5 (64.1, 67.0)	78.8 (78.5, 79.1)
	20-39	109,677	43.3 (42.7, 43.9)	47.4 (47.2, 47.5)	59.2 (59.1, 59.2)
	40-59	88,509	25.2 (24.6, 25.7)	30.2 (30.1, 30.3)	40.0 (40.0, 40.1)
	60-79	32,679	12.6 (12.2, 13.1)	16.0 (16.0, 16.1)	22.7 (22.6, 22.7)
	80+	3,822	4.0 (4.0, 4.0)	5.9 (5.9, 5.9)	9.4 (9.4, 9.4)
Pacific Peoples	Males				
	0-19	60,825	54.7 (51.8, 57.6)	66.4 (65.2, 67.6)	79.2 (78.8, 79.5)
	20-39	47,028	37.6 (36.1, 39.2)	47.9 (47.6, 48.1)	59.6 (59.5, 59.7)
	40-59	33,477	21.9 (20.3, 23.4)	32.1 (32.0, 32.2)	40.7 (40.6, 40.8)
	60-79	11,823	14.2 (13.1, 15.4)	17.2 (17.1, 17.3)	23.0 (22.9, 23.1)
	80+	975	3.5 (3.5, 3.5)	5.7 (5.7, 5.7)	9.3 (9.3, 9.3)
	Females				
	0-19	58,410	65.4 (64.5, 66.2)	73.7 (73.5, 73.8)	82.9 (82.7, 83.1)
	20-39	45,261	45.4 (44.5, 46.2)	53.7 (53.5, 53.8)	63.2 (63.1, 63.2)
	40-59	33,426	27.1 (26.3, 27.9)	35.5 (35.3, 35.6)	43.8 (43.7, 43.9)
	60-79	12,834	10.6 (10.2, 11.0)	20.0 (19.9, 20.1)	25.5 (25.5, 25.6)
	80+	1,773	1.2 (1.2, 1.2)	8.2 (8.2, 8.2)	10.3 (10.3, 10.3)

Over the 5-year study period, 156,099 (3.5%) of participants died. Higher death rates were seen for those with T1D (7.4%) and T2D (14.8%) over the study period than for those without diabetes (2.8%). Māori and Pacific Peoples with T2D had lower mortality rates than Europeans/Others with T2D (14.4% vs 9.6% vs 18.4% respectively). Lower death rates for Māori and Pacific Peoples with T1D were also observed as described in Table 4.

**Table 4:** Observed 5-year mortality events for those with DM, stratified by ethnicity and DM sub-type.

Ethnicity	T1D Deaths		T2D Deaths		Deaths Without Diabetes	
	*n*	%	*n*	%	*n*	%
European/Other	972	7.9	25,806	18.4	99,945	3.7
Māori	150	7.2	6,273	14.4	12,255	1.6
Pacific Peoples	36	6.1	2,769	9.6	3,027	1.1
Asian	21	2.0	1,884	5.2	2,955	0.6
Total	1,179	7.4	36,732	14.8	118,182	2.8

With the exception of deaths from diseases of the nervous system, every category that was able to be analysed without suppressed data demonstrated higher mortality rates for those with T1D and T2D compared to those without DM (Table 5). For both T1D and T2D, diseases of the circulatory system accounted for the largest proportion of deaths (26.7% and 34.7% respectively) followed by neoplasms (20.6% and 24.9% respectively).

**Table 5:** Cause-specific mortality rates per 100,000 people for those with T1D, T2D, and without diabetes.

Cause of Death (ICD-10-AM Chapters)	T1D Deaths			T2D Deaths			Deaths without Diabetes		
	*n*	%	Deaths per 100,000	*n*	%	Deaths per 100,000	*n*	%	Deaths per 100,000
Certain Infections and Parasitic Diseases	12	1.0	75.3	396	1.1	159.6	1,005	0.9	23.7
Neoplasms	243	20.6	1524.3	9,147	24.9	3686.6	3,713	3.1	875.4
Solid Neoplasms	210	17.8	1317.3	8,427	22.9	3396.4	3,416	2.9	805.4
Liquid Neoplasms	27	2.3	169.4	480	1.3	193.5	2,151	1.8	50.7
Blood and Blood-Forming Organs and Certain Disorders Involving the Immune Mechanism	Suppressed			81	0.2	32.6	288	0.2	6.8
Endocrine, Nutritional and Metabolic Diseases	375	31.8	2352.3	4,206	11.5	1695.2	1,068	0.9	25.2
Type 1 diabetes mellitus	216	18.3	1354.9	33	0.1	13.3	Suppressed		
Coma/ketoacidosis	42	3.6	263.5	Suppressed			Suppressed		
Type 2 diabetes mellitus	138	11.7	865.6	3,735	10.2	1505.4	87	0.1	2.1
Coma/ketoacidosis	6	0.5	37.6	54	0.1	21.8	9	0.0	0.2
Diabetic nephropathy	126	10.7	790.4	1,530	4.2	616.6	24	0.0	0.6
Mental and Behavioural Disorders	42	3.6	263.5	1,908	5.2	769.0	7,989	6.8	188.4
Nervous System	18	1.5	112.9	1,212	3.3	488.5	6,492	5.5	153.1
Circulatory System	315	26.7	1975.9	12,762	34.7	5143.6	36,093	30.5	850.9
Ischaemic heart disease	177	15.0	1110.3	6,648	18.1	2679.4	16,146	13.7	380.7
Cerebrovascular disorders	69	5.9	432.8	2,592	7.1	1044.7	8,763	7.4	206.6
Respiratory System	51	4.3	319.9	3,126	8.5	1259.9	11,484	9.7	270.7
Digestive System	33	2.8	207.0	1,236	3.4	498.2	3,651	3.1	86.1
Skin and Subcutaneous	Suppressed			189	0.5	76.2	402	0.3	9.5
Musculoskeletal System and Connective Tissue	Suppressed			270	0.7	108.8	912	0.8	21.5
Genitourinary System	15	1.3	94.1	678	1.8	273.3	1,611	1.4	38.0
Congenital Malformations, Deformations and Chromosomal Abnormalities	Suppressed			51	0.1	20.6	507	0.4	12.0
External Causes of Morbidity and Mortality	54	4.6	338.7	1,206	3.3	486.1	8,400	7.1	198.0

## Discussion

The current study provides a contemporary overview of diabetes in AoNZ, including LE effects, population distributions, and associated mortality rates. Our dataset provides a clear demonstration of the negative impact on estimated remaining LE years that a diagnosis of diabetes confers, with an age-dependent reduction in remaining LE years seen in all age groups. Also of note, in both men and women, T1D was associated with fewer estimated remaining LE years than T2D, and men with DM from early childhood had lower remaining LE years than women. On a population level, those with T2D had nearly five-fold higher death rates than the general population.

Considerable ethnic differences in remaining LE years were also evident during sub-group analysis. For example, for those aged less than 19 with T1D, Māori women had an 18.0 year reduction in estimated remaining LE years compared to Māori women without DM, while 12.4 and 17.5 remaining LE year reductions were seen in the overall and Pacific samples respectively. In comparison, for the overall sample, men and women aged 0–19 years with T1D saw a 13 and 12.4 year reduction in remaining LE respectively. These findings suggest a disproportionate negative LE impact of DM in these groups.

Previous data suggest that both Māori and Pacific Peoples have a reduced LE compared to the overall population and that that diabetes is responsible for 0.4–0.7 years of this LE differential, while our data suggest that Pacific Peoples without DM have similar remaining LE years compared to the total sample across all age groups [37,38]. However, the disproportionate LE loss in Māori participants with DM occurs in the context of an already lower remaining LE, earlier age of onset of T2D, and higher rates of cardiovascular and renal complications even for those without DM [39]. In the global context, while diabetes is implicated as a risk factor for adverse outcomes in many indigenous groups, publications with exact quantification of the impact of diabetes on remaining LE years, as found in this analysis, appear to be limited [40–43]. While population-specific analyses of DM and remaining LE data are available for several countries, there has previously been a paucity of remaining LE data focusing on outcomes for indigenous persons.

In our analysis, LE loss for the overall population is somewhat different to that described in other countries where white ethnicity predominates. For example, AoNZ men and women with diabetes aged 50–54 years, had a 5.6 and 6.0 year reduction in estimated remaining LE years respectively which is higher than Australians with diabetes aged 50 (3.2 and 3.1 year loss in men and women respectively) but lower than 50-year-old participants in the Framingham Heart Study (7.5 year and 8.2 year reduction for men and women respectively) [44,45]. AoNZ men and women with T1D aged 20–39 years had a 12.1 and 11.6 year LE loss respectively, compared to a 10.1–11.1 and 11.3–12.9 year LE difference in Scottish men and women in this age band respectively [46]. Such variations are likely multi-factorial, including different healthcare delivery systems, different sociodemographic structures, and variations in study designs used to calculate these outcomes. The overall prevalence of T1D and T2D (0.4% and 5.5% respectively) are in keeping with some international data, but higher than reported in other publications [47–49]. The dataset also provides contemporary insights into diabetes prevalence in Māori and Pacific Peoples, with the current analysis indicating somewhat lower estimates than in previous reports [50].

As can be anticipated, the estimated remaining LE years is inversely related to increasing age group category. Furthermore, with increasing age, the difference between estimated remaining LE years becomes less marked in people with diabetes versus without diabetes. This obviously reflects the increasing incidence of other mortality drivers as age progresses but may also suggest the ‘drop out’ from the sample of those with poorly controlled DM who may have a younger mortality. This is in keeping with a previous meta-analysis indicating a 4% reduction in all-cause mortality risk per one year increase in age of diabetes diagnosis [51]. A previous AoNZ analysis has also demonstrated a near four-fold increase in mortality in children or young-adults with T1D, while another investigation indicated a 33% reduction in relative hazard for mortality for each additional 10-year increment in age of onset [14,52]. Of course, the earlier death of some with DM (which is more likely if diagnosed at a young age as seen with our data) may introduce a survivorship bias when analysing for causes of death at a wider population level, for example as seen with reductions in hazard ratios for adverse cardiovascular outcomes and all-cause mortality with increasing DM age [53]. Our analysis has also demonstrated the older age of those with DM (median age 27 years higher than those without DM) which is also anticipated to be a significant driver for the increased mortality rates seen. The higher unadjusted mortality rates for Europeans demonstrated in this study also likely reflects the older average age in this demographic. The effect modification of diabetes on mortality risk across different age groups is the subject of a current investigation by our team.

Increases in LE for people with diabetes has led to a large increase in the number of years spent with diabetes for the average person, potentially increasing time spent disabled or with additional morbidity; described as the ‘morbidity expansion’ concept [2,5]. The Type 1 Diabetes Index estimates 22 years of healthy years of life lost per person with T1D in AoNZ, while older persons with DM also see a reduction in the ability to perform basic care tasks required for independent living (active life expectancy) [15,54]. The current analysis has not captured any quality of life data to further explore this important part of having a chronic condition.

While certain cause of death categories were unable to be analysed due to low participant numbers, predominantly in the T1D group, the current data implicated DM as a risk factor for mortality in nearly all categories of death that were able to be analysed. While endocrine, metabolic, cardiovascular, and neoplastic causes of death are well-described in the setting of DM, our data, which also shows increased mortality in categories such as Mental and Behavioural Disorders and External Causes, are in keeping with other publications that highlight the far-reaching impacts that DM confers [55,56]. Our findings of circulatory disease accounting for 26.7 and 34.7% of T1D and T2D deaths is somewhat lower than previous AoNZ and international estimates.

The current cause of death categories were not refined to a degree to detect potential subtle variations. For example, a 2010 consensus report indicates that DM is a risk factor for some cancers (such as liver, pancreas, endometrium), while others (prostate) have a reduced risk – whereas our analysis has grouped all solid malignancies together [57]. The current analysis also did not evaluate how causes of death of those with DM evolve with age group. International data indicate an evolution from endocrine and metabolic causes of morbidity and mortality in younger patients, to circulatory and renal disease in older age groups [46,58,59]. Of note, differences in mortality rates by ethnicity were also not currently analysed, though differences in cause-specific mortality have previously been demonstrated [9]. Finally, under-documentation of diabetes is estimated in up to 50% of AoNZ death certificates, thereby affecting the reliability of mortality data [60].

The current analysis included further limitations. Without the use of prioritised ethnicity outputs, Māori have previously been under-represented in several data sets. However, this approach is limited in its inability to cater for those identifying with more than one ethnic group and a converse biasing effect arising from the under representation of Pacific Peoples, and over-representation of other groups, in pursuant analyses may arise [32,61,62]. The VDR is also limited in that it does not contain diagnostic data explicitly delineating between T1D and T2D (or other rarer DM subtypes), therefore, requiring the application of external algorithms to generate these categories. These algorithms, however, have been demonstrated to generate false-positive and-negative classifications but, in the AoNZ setting, currently remain the most effective approach for distinguishing T1D and T2D at a population level [63]. This miscoding of patients, in the absence of cross-referencing to other databases, may skew subsequent analyses based on VDR data.

Mortality analysis was also subject to potential data coding errors, for example as was demonstrated by the presence of T2D as a cause of death in those identified as having either T1D or no diagnosis of DM. Of note, the identification of participants with DM was binary, and no further analysis by risk modifiers such as duration of diabetes, average HbA1c, blood pressure profiles, dyslipidaemia, or fasting blood sugar levels, was possible. At present, the VDR does not store population-level data for these additional risk predictors and can, therefore, limit the capacity to attribute mortality differences to diabetes alone. Different cause-specific mortality rates may be seen in those with shorter duration or well-controlled DM in comparison to longer duration or less controlled participants. The present analysis also treated sex as a binary input owing to the very small number of participants identified as non-binary during the study window. The impact of concurrent risk factors that also influence mortality for people with diabetes was also not evaluated [64].

Of note, the introduction of publicly-funded Sodium-Glucose Transport Protein 2 Inhibitors and Glucagon-Like Peptide-1 Receptor Agonists only occurred in AoNZ in 2021, after the current study window. Access criteria for these agents had an equity-driven focus, with access more readily available for Māori and Pacific Peoples. Early data since this introduction do point to increased prescription uptakes for Māori and Pacific Peoples [65,66]. It remains to be seen, however, whether the increased uptake will eventually be reflected in improved and more equitable clinical outcomes.

## Conclusion

This is the first study in more than a decade, using AoNZ-derived data to evaluate the LE impacts for New Zealanders with DM across various age-groups, and the first comparing T1D and T2D outcomes within the same analysis. Of note, the current study provides detailed outcomes for Māori and Pacific Peoples. We quantify the markedly skewed remaining LE expectancy effects for Māori and Pacific Peoples with DM compared to other ethnic groups. Our data also provides confirmation of the wide-reaching mortality risk that accompanies a diagnosis of DM, and provides comparisons of mortality rates between those diagnosed with T1D and T2D by cause of death category. Despite global advances in diabetes care and available treatment modalities, our findings highlight the importance for ongoing research and equity-focussed healthcare strategies to optimise outcomes for people with DM, particularly those diagnosed in earlier years, and Māori and Pacific Peoples.

## Supporting information

S1 Table Distribution of DM diagnoses, stratified by age group, sex and DM subtype. (PDF)

S2 Table Abridged period life table for people with diabetes vs without diabetes stratified by sex and 5-year age band. (PDF)
