# Supplementary material for: Life expectancy and mortality of people with and without diabetes in Aotearoa | New Zealand: A national cohort study
Source: PLoS One. 2026 May 4;21(5):e0345892. doi: 10.1371/journal.pone.0345892 (PMC13138630; doi:10.1371/journal.pone.0345892)
Supplement: S1 Table — (PDF) [file pone.0345892.s001.pdf]

**S1 Table: Distribution of DM diagnoses, stratified by age group, sex and DM subtype.**

| Age group (years) | T1D | | T2D | | Without Diabetes | |
|---|---|---|---|---|---|---|
| | n | % | n | % | n | % |
| Men | | | | | | |
| 0-19 | 1,188 | 15·4 | 1,092 | 0·9 | 617,007 | 29·3 |
| 20-39 | 2,238 | 29·0 | 6,330 | 5·1 | 595,086 | 28·3 |
| 40-59 | 2,472 | 32·0 | 42,468 | 34·0 | 548,844 | 26·1 |
| 60-79 | 1,572 | 20·4 | 61,545 | 49·3 | 290,868 | 13·8 |
| 80+ | 246 | 3·2 | 13,521 | 10·8 | 50,736 | 2·4 |
| Women | | | | | | |
| 0-19 | 1,110 | 13·5 | 1,056 | 0·9 | 584,601 | 27·3 |
| 20-39 | 3,111 | 37·8 | 11,256 | 9·1 | 576,624 | 27·0 |
| 40-59 | 2,433 | 29·6 | 39,621 | 32·2 | 576,642 | 27·0 |
| 60-79 | 1,302 | 15·8 | 54,450 | 44·2 | 321,231 | 15·0 |
| 80+ | 270 | 3·3 | 16,770 | 13·6 | 79,785 | 3·7 |
