# Supplementary material for: Life expectancy and mortality of people with and without diabetes in Aotearoa | New Zealand: A national cohort study
Source: PLoS One. 2026 May 4;21(5):e0345892. doi: 10.1371/journal.pone.0345892 (PMC13138630; doi:10.1371/journal.pone.0345892)
Supplement: S2 Table — (PDF) [file pone.0345892.s002.pdf]

S2 Table: Abridged period life table for people with diabetes vs without diabetes stratified by sex.

Age group (years)	Diabetes N	Estimated remaining life expectancy (years)					
		With diabetes		Without diabetes		Difference	
		x̄	(95% CI)	x̄	(95% CI)	x̄	(95% CI)
Men							
0-4	155,529	69·7	(69·6, 69·8)	79·4	(79·4, 79·4)	9·7	(9·6, 9·8)
5-9	157,983	64·7	(64·6, 64·8)	74·5	(74·5, 74·5)	9·7	(9·6, 9·9)
10-14	148,683	60·0	(59·9, 60·1)	69·5	(69·5, 69·5)	9·5	(9·4, 9·7)
15-19	157,092	55·0	(54·9, 55·1)	64·6	(64·6, 64·6)	9·6	(9·5, 9·7)
20-24	172,746	50·6	(50·5, 50·6)	59·8	(59·8, 59·8)	9·3	(9·2, 9·4)
25-69	155,430	46·1	(46·0, 46·2)	55·0	(55·0, 55·1)	9·0	(8·9, 9·1)
30-34	141,147	42·0	(42·0, 42·1)	50·2	(50·2, 50·3)	8·2	(8·1, 8·3)
35-39	134,331	37·9	(37·9, 38·0)	45·4	(45·4, 45·5)	7·5	(7·4, 7·6)
40-44	150,912	33·8	(33·7, 33·8)	40·7	(40·6, 40·7)	6·9	(6·9, 7·0)
45-49	152,259	29·5	(29·5, 29·6)	36·0	(35·9, 36·0)	6·4	(6·4, 6·5)
50-54	154,626	25·7	(25·7, 25·7)	31·3	(31·3, 31·4)	5·6	(5·6, 5·7)
55-59	135,987	21·9	(21·9, 22·0)	26·9	(26·8, 26·9)	4·9	(4·9, 5·0)
60-64	117,630	18·3	(18·3, 18·3)	22·5	(22·5, 22·6)	4·2	(4·2, 4·3)
65-69	106,131	14·9	(14·9, 15·0)	18·4	(18·4, 18·4)	3·5	(3·4, 3·5)
70-74	76,491	11·7	(11·6, 11·7)	14·5	(14·4, 14·5)	2·8	(2·7, 2·8)
75-79	53,736	8·8	(8·8, 8·9)	11·0	(10·9, 11·0)	2·2	(2·1, 2·2)
80-84	36,309	6·5	(6·4, 6·5)	7·9	(7·9, 8·0)	1·4	(1·4, 1·5)
85-89	20,193	4·7	(4·6, 4·7)	5·7	(5·6, 5·7)	1·0	(0·9, 1·0)
90-94	6,792	3·5	(3·4, 3·5)	4·2	(4·2, 4·2)	0·7	(0·6, 0·7)
95-99	1,083	2·8	(2·7, 2·9)	3·5	(3·5, 3·6)	0·7	(0·6, 0·7)
100+	123	†		†		†	
Women							
0-4	147,102	74·2	(74·1, 74·3)	82·8	(82·8, 82·8)	8·6	(8·5, 8·7)
5-9	149,607	69·2	(69·1, 69·3)	77·9	(77·8, 77·9)	8·7	(8·6, 8·8)
10-14	141,612	64·4	(64·3, 64·5)	72·9	(72·9, 72·9)	8·5	(8·4, 8·6)
15-19	148,446	59·8	(59·7, 59·8)	68·0	(67·9, 68·0)	8·2	(8·1, 8·3)
20-24	158,097	55·2	(55·1, 55·3)	63·1	(63·0, 63·1)	7·9	(7·8, 7·9)
25-69	150,225	50·4	(50·4, 50·5)	58·2	(58·1, 58·2)	7·7	(7·7, 7·8)
30-34	143,328	45·9	(45·8, 45·9)	53·3	(53·2, 53·3)	7·4	(7·3, 7·4)
35-39	139,341	41·2	(41·2, 41·3)	48·4	(48·4, 48·4)	7·2	(7·1, 7·2)
40-44	159,045	36·7	(36·6, 36·7)	43·5	(43·5, 43·5)	6·9	(6·8, 6·9)
45-49	158,445	32·2	(32·2, 32·3)	38·8	(38·7, 38·8)	6·5	(6·5, 6·6)
50-54	160,677	28·1	(28·1, 28·2)	34·1	(34·0, 34·1)	6·0	(5·9, 6·0)
55-59	140,532	24·1	(24·1, 24·1)	29·5	(29·4, 29·5)	5·4	(5·3, 5·4)
60-64	122,199	20·2	(20·2, 20·2)	25·0	(24·9, 25·0)	4·7	(4·7, 4·8)
65-69	110,409	16·5	(16·5, 16·6)	20·6	(20·6, 20·6)	4·0	(4·0, 4·1)
70-74	82,374	13·1	(13·1, 13·1)	16·4	(16·4, 16·4)	3·3	(3·3, 3·3)
75-79	62,004	10·0	(10·0, 10·1)	12·5	(12·5, 12·6)	2·5	(2·5, 2·5)
80-84	46,530	7·3	(7·3, 7·3)	9·1	(9·1, 9·1)	1·8	(1·8, 1·8)
85-89	31,551	5·3	(5·2, 5·3)	6·4	(6·4, 6·4)	1·1	(1·1, 1·1)
90-94	14,898	3·8	(3·8, 3·9)	4·5	(4·5, 4·6)	0·7	(0·6, 0·7)
95-99	3,396	3·0	(2·9, 3·0)	3·3	(3·3, 3·2)	0·3	(0·3, 0·3)

100+	453	†	†	†

† Unable to be calculated
